# Sputter-Deposited Binder-Free Nanopyramidal Cr/γ-Mo_2_N TFEs for High-Performance Supercapacitors

**DOI:** 10.1186/s11671-022-03704-5

**Published:** 2022-07-19

**Authors:** Durai Govindarajan, Nithyadharseni Palaniyandy, Karthik Kumar Chinnakutti, Mai Thanh Nguyen, Tetsu Yonezawa, Jiaqian Qin, Soorathep Kheawhom

**Affiliations:** 1 Department of Chemical Engineering, Faculty of Engineering, Chulalongkorn University, Bangkok, 10330 Thailand; 2 Institute for the Development of Energy for African Sustainability, College of Engineering, Science and Technology, University of South Africa, Florida Science Campus, Roodepoort, 1709 South Africa; 3 Department of Chemistry, Vinayaka Mission’s Kirupananda Variyar Arts and Science College, Vinayaka Mission’s Research Foundation (Deemed to be University), 636308, Salem, India; 4 Division of Materials Science and Engineering, Faculty of Engineering, Hokkaido University, Hokkaido, 060-8628 Japan; 5 Metallurgy and Materials Science Research Institute, Chulalongkorn University, Bangkok, 10330 Thailand; 6 Center of Excellence on Advanced Materials for Energy Storage, Chulalongkorn University, Bangkok, 10330 Thailand; 7 Bio-Circular-Green-economy Technology & Engineering Center (BCGeTEC), Faculty of Engineering, Chulalongkorn University, Bangkok, 10330 Thailand

**Keywords:** Molybdenum nitride, Chromium-doped, Co-sputtering, Supercapacitors, Cycling stability

## Abstract

Due to their outstanding power density, long cycle life and low cost, supercapacitors have gained much interest. As for supercapacitor electrodes, molybdenum nitrides show promising potential. Molybdenum nitrides, however, are mainly prepared as nanopowders via a chemical route and require binders for the manufacture of electrodes. Such electrodes can impair the performance of supercapacitors. Herein, binder-free chromium (Cr)-doped molybdenum nitride (Mo_2_N) TFEs having different Cr concentrations are prepared via a reactive co-sputtering technique. The Cr-doped Mo_2_N films prepared have a cubic phase structure of γ-Mo_2_N with a minor shift in the (111) plane. While un-doped Mo_2_N films exhibit a spherical morphology, Cr-doped Mo_2_N films demonstrate a clear pyramid-like surface morphology. The developed Cr-doped Mo_2_N films contain 0–7.9 at.% of Cr in Mo_2_N lattice. A supercapacitor using a Cr-doped Mo_2_N electrode having the highest concentration of Cr reveals maximum areal capacity of 2780 mC/cm^2^, which is much higher than that of an un-doped Mo_2_N electrode (110 mC/cm^2^). Furthermore, the Cr-doped Mo_2_N electrode demonstrates excellent cycling stability, achieving ~ 94.6% capacity retention for about 2000 cycles. The reactive co-sputtering proves to be a suitable technique for fabrication of binder-free TFEs for high-performance energy storage device applications.

**Graphical Abstract:**

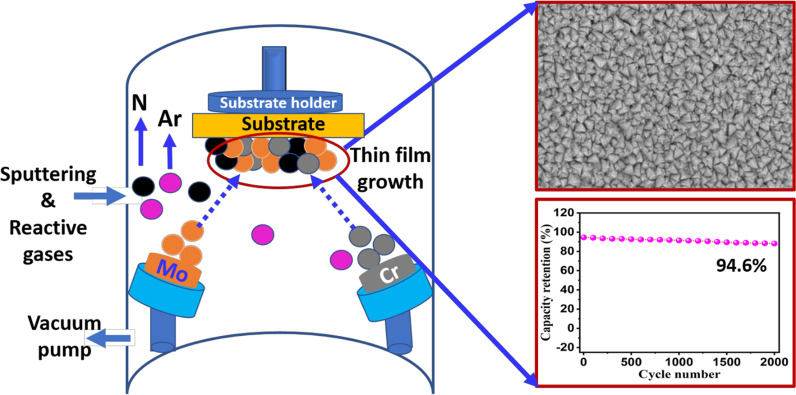

**Supplementary Information:**

The online version contains supplementary material available at 10.1186/s11671-022-03704-5.

## Introduction

Electrochemical capacitors (ECs) or supercapacitors (SCs) are in high demand for a variety of applications, e.g., portable, wearable/flexible devices and electronic industries, as well as electric vehicles (EVs) due to their high-power density, fast charge–discharge and longer cycle life, [1–4]. Because of their enormous surface area, carbon-based materials (EDLC) including activated carbon remain the most used electrode material for ECs. However, carbon-based materials exhibit inferior energy density [5, 6]. Owing to their high capacitance, metal oxides/nitrides (pseudocapacitors) have been explored as alternative electrode materials for batteries and supercapacitors [5–8]. Yet, metal oxides reveal low electrical conductivity, which restricts their capacitance/capacity. There has been an urgent need, therefore, to replace carbon and metal oxide-based electrode materials with high-performance electrode materials [9].

Recently, sulfides and nitride-based materials have shown promise for the development of high-performance electrochemical ECs due to their outstanding electrochemical properties [10–12]. Because of their structural stability, unique physiochemical features, good electrocatalytic activities and superior electrical conductivity, metal nitrides are highly considered electrode material for ECs [12, 13]. So far, nitride-based electrodes, such as TiN [14, 15], VN [16–19], RuN [20], Mo_2_N [21–23], Ni_2_Mo_3_N [24], Ni_3_N [25], MnN@rGO [26] and CrN [27–29] electrodes, have been investigated for application in ECs. Molybdenum nitrides have been pursued as a viable electrode material for ECs owing to their great catalytic activity, excellent electrochemical behavior, low compressibility and high melting point. However, molybdenum nitrides as well as other metal nitrides are mainly prepared using chemical synthesis techniques, which result in mechanical instability and significant energy consumption. Moreover, studies of their electrochemical properties are still inadequate [30–32]. Nanomaterials prepared by chemical synthesis techniques in the form of nanopowder are in need of binders such as polyvinylidene fluoride (PVDF), polyvinyl alcohol (PVA) and carboxymethyl cellulose (CMC) to fabricate electrodes. Yet, such binders are known to hinder the performance of electrodes. Therefore, researchers are searching for other synthesis methods without using any binder [10, 25].

Due to the uniformity plus controlled stoichiometry of their coatings, good adhesion and well-defined structure, binder-free nitride-based thin film electrodes (TFEs) prepared by a sputtering technique are noted for producing high-performance, stable and flexible ECs [30]. Of note, the [111] grown molybdenum nitride films grown by using reactive direct current (DC) magnetron sputtering on titanium substrate at 400 °C demonstrate high areal capacitance of 55 mF cm^−2^ and excellent cycling stability of 100% capacitance retention after 2000 cycles [33]. Adalati et al. [34] synthesized a molybdenum and vanadium nitride binder-free thin film on a stainless steel substrate through reactive sputtering technique by varying the sputtering parameters such as Ar/N_2_ gas flow, applied DC power and deposition pressure. Vanadium nitride and molybdenum nitride were used to develop an asymmetrical device. These electrodes showed areal capacitances of 82.35 mF cm^−2^ (MoN) and 67.50 mF cm^−2^ (VN), respectively, with a capacitance retention of approximately 95.23%. Shi et al. [35] synthesized intercolumnar porous CrN TFEs at a substrate temperature of 250 °C under various N_2_ ratios, which displayed an areal capacitance of 41.7 mF cm^−2^ in H_2_SO_4_ electrolyte. Gao et al. [36] reported the synthesis of nanoporous CrN containing different ratios of metallic nickel (Ni), viz. 0, 30.4, 54.2 and 77.6 at.% (CrN-Ni) using arc ion plating. The nanoporous coating obtained using 54.2 at.% Ni, containing the CrN-Ni film, exhibited the highest capacitance of 58.5 mF cm^−2^ at 1.0 mA cm^−2^ greater than all other coatings, i.e., much higher than that of the as-deposited CrN electrode. In addition, this binder-free electrode provided an excellent capacitance retention rate. In general, doping of selective metal ions into host materials has proved to be a very good way to increase electrical conductivity and capacitance. However, there is still a gap in the development of binder-free metal nitride-based electrodes. To the best of our knowledge, no report has been published previously on the synthesis of binder-free Cr-doped Mo_2_N TFEs (TFEs) material for ECs. Thus, for the first time, novel binder-free Cr-substituted Mo_2_N TFEs have been developed for high-efficiency energy storage devices; their improved electrochemical performances are compared with existing metal nitrides-based electrodes.

Cr was chosen as a dopant in this work mainly because it is a metal having good electrical conductivity (0.0774 10^6^/cm Ω and 7.9 × 10^6^ S/m) and low ionic radii (0.62 Å). Cr is low in cost compared with other high conductive metals (Ag, Pt, V, Ru Ti and Ni). Besides, Cr is abundant (83% natural abundance) as well as corrosion-resistant. The addition of Cr in Mo_2_N synergistically alters the electronic states and creates better attainable active sites, enhancing the electrochemical performance and improving conductivity.

In the present work, nanopyramidal Cr-doped Mo_2_N (Cr/Mo_2_N) binder-free TFEs with different Cr doping concentrations: 0 to ~ 8 at.%, have been successfully synthesized via a reactive magnetron co-sputtering method for high-performance energy storage devices. Binder-free Mo_2_N TFEs show promise as active anode material for ECs. The impact of Cr on Mo_2_N as well as the microstructural and electrochemical charge storage properties of binder-free TFEs is discussed.

## Experimental Section

### Cr-Doped Mo_2_N Thin Films Deposition

**Fig. 1:**
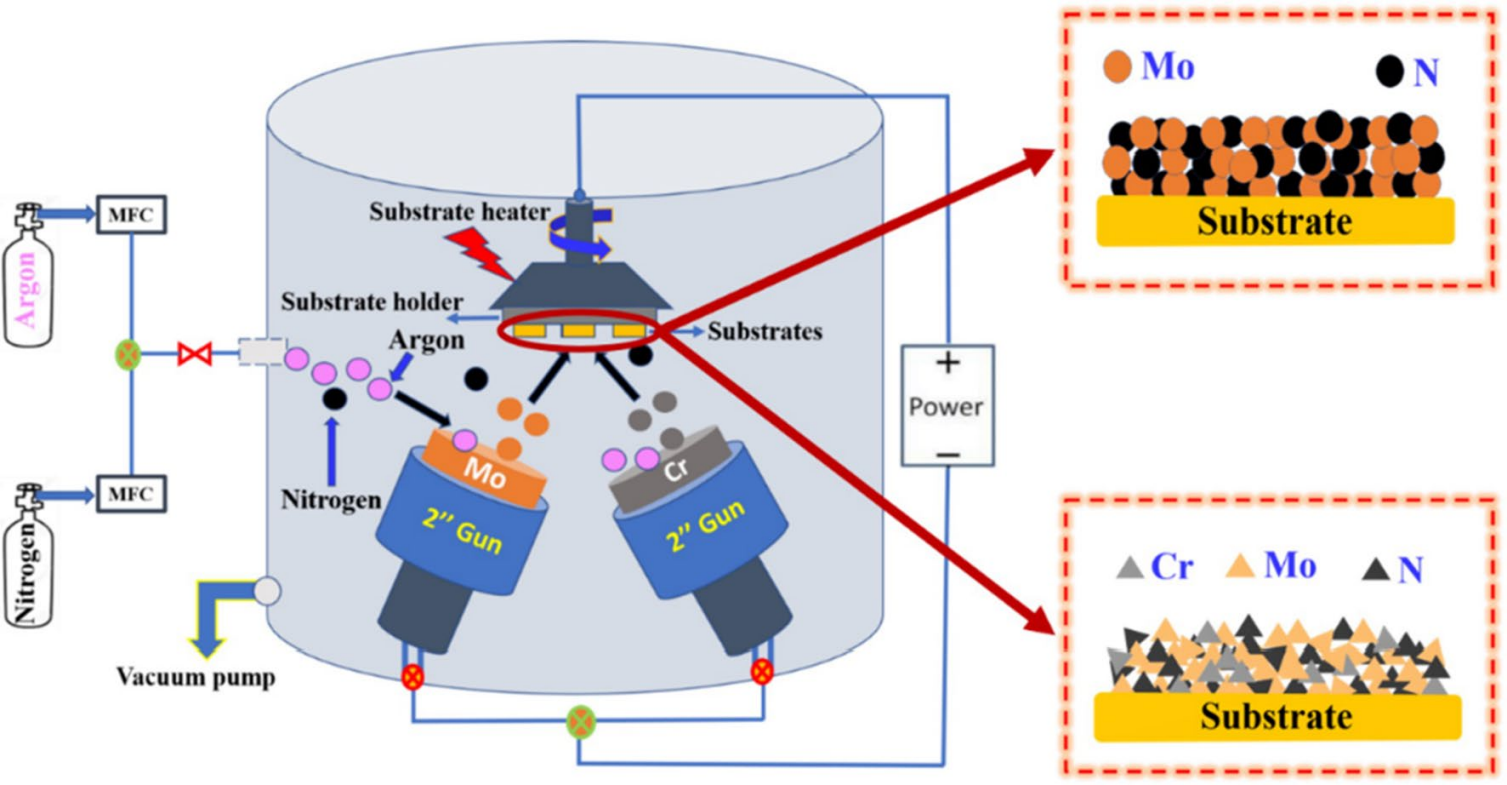
Schema of the Cr-doped Mo_2_N thin films using a reactive co-sputtering process

Cr-doped MoN thin films are synthesized via a reactive co-sputtering technique (MP 300 sputter system, Plassys, France) along with 2 inch Cr and Mo targets having a purity of ~ 99.99% using argon (Ar^+^) as sputtering and nitrogen (N_2_) as reactive gases. In advance of deposition, the substrates: glass, silicon (100) and stainless steel 304, were cleaned (ultrasonically) by a standard cleaning process using acetone/ethanal and deionized water to eliminate the native oxide layers or any other impurities on the surface of the substrates. Subsequently, the substrates were dried and loaded into a sputtering chamber, as shown in Fig. 1.

After loading the substrates into the sputtering chamber, a base vacuum of 4 × 10^−6^ mbar was achieved using a turbo-molecular pump. Initially, before conducting deposition, both metallic targets were pre-sputtered in the Ar^+^ environment for ~ 10 min to eliminate residual native oxides over the target surfaces. During the growth of the film, a negative charge is applied to the target material (Mo and Cr) to initiate sputtering, which ionizes the working gas of the Ar^+^ and N sources. Positively charged Ar ions, generated in the plasma region, are rapidly attracted to the negatively biased Mo and Cr targets. Consequently, the atomic-sized Mo and Cr particles are ejected from the targets (Mo and Cr) as a result of the collision's momentum transfer. The plasmas negatively charged N_2_ atoms react with the Cr and Mo atoms; the resultant Cr/MoN thin film is ultimately deposited on the surface of the substrates. Herein, the Cr atoms introduced bind to the Mo_2_N crystal lattice directly through a three-body collision of Cr, Mo and N_2_ (Additional file 1: Fig. S1). All the films were prepared at a substrate temperature of 573 K (± 5) and working pressure of 9.8 mTorr. The Cr-doped Mo_2_N thin films were synthesized by fixing the Mo sputter power and varying Cr target power having a constant Ar/N_2_ flow rate. For the deposition of the pure Mo_2_N sample preparation, Mo target power remained unchanged. In Table 1, the detailed deposition parameters of this work are given. The un-doped Mo_2_N denotes the Mo_2_N film having 0 at.% of Cr. In addition, Cr/Mo_2_N-1, Cr/Mo_2_N-2, Cr/Mo_2_N-3 and Cr/Mo_2_N-4 denote the Cr-doped Mo_2_N films containing 3.35, 4.87, 6.21 and 7.90 at.% Cr, respectively.

**Table 1:** Sputtering parameters for the development of Cr-doped Mo_2_N thin films

Sputtering deposition parameters	Range
Sputter targets	Metallic molybdenum (Mo) and chromium (Cr) targets: 99.99% purity
Substrates	Type 304 SS and silicon (100)
Target-to-substrate distance	5–6 cm
Base pressure	4 × 10^−6^ mbar
Working pressure	9.8 mTorr (1.30 × 10^−2^ mbar)
Substrate temperature	573 K
Sputtering gas (Ar)	14 sccm
Reactive sputtering gas (N_2_)	2 sccm
Mo target power (RF gun)	100 (± 2) W
Cr target power (DC gun)	0, 8, 10, 12 and 14 (± 2) W (for doping)
Duration of the deposition	60 min

### Characterization Techniques

The sputter-deposited Cr-doped Mo_2_N thin films were investigated using various characterization techniques. Surface morphology and elemental composition of the Cr-doped Mo_2_N films were examined by field emission scanning electron microscope (FE-SEM, Carl Zeiss, Supra 55, Germany) and equipped with energy-dispersive X-ray spectrometer (EDS, Oxford instrumental), respectively. The crystallographic orientation and phase purity of the Cr-doped Mo_2_N films were characterized using grazing incidence X-ray diffractometer (XRD, D8 Advance, Bruker, Germany). XRD patterns were recorded at the diffraction angle of 2*θ* = 20°–70° using the Cu-K*α* radiation wavelength (*λ* = 1.54 Å). To understand the oxidation states and chemical/electronic configuration of the thin films, X-ray photoelectron spectroscopy (XPS) technique using a ULVAC-PHI, Inc. (PHI Quantera SXM, USA) with an Al K_*α*_ X-ray source was adopted.

### Electrochemical Measurements

Electrochemical behavior of the Cr-doped Mo_2_N TFEs was assessed using an electrochemical workstation (Bio-logic, SP-300, France) in a 3-electrode cell configuration in 1 M KOH electrolyte. The as-deposited Mo_2_N and Cr-doped Mo_2_N films on the stainless steel substrates were directly utilized as working electrodes. Ag/AgCl and platinum wire were used as reference and counter electrodes, respectively. Both cyclic voltammetry (CV) and galvanostatic charge–discharge (GCD) techniques were applied to calculate the areal capacity/capacitance with respect to different scan rates and current densities as well as the stability performance of the TFEs. The electrochemical impedance spectroscopy (EIS) technique was employed to examine the charge transfer mechanism of the film electrodes during electrochemical analysis.

## Results and Discussion

### Structural Characterization

**Fig. 2:**
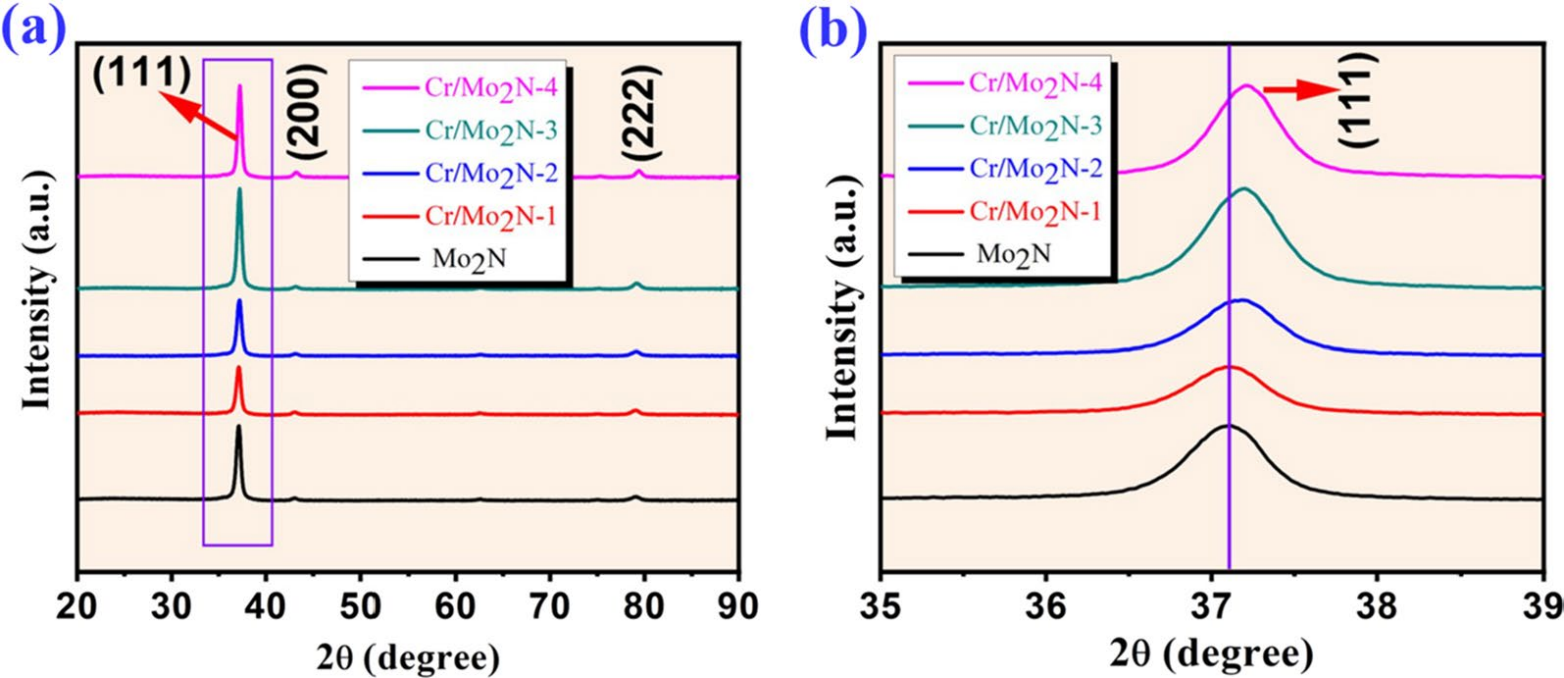
**a** XRD patterns of Mo_2_N and Cr-doped Mo_2_N and **b** enlargement of the peak shift of (111) plane with respect to the doping concentration of Cr

Phase formation and crystal structure of the Mo_2_N and Cr-doped Mo_2_N thin films with thicknesses ranging from ~ 900 to 1400 nm (Additional file 1: Fig. S2) were initially investigated by XRD analysis. During XRD analysis, scan limit was fixed in the 2*θ* range of 20°–80°. In Fig. 2, the XRD patterns of as-deposited Mo_2_N and Cr-doped Mo_2_N thin films are displayed.

In both the un-doped and Cr-doped Mo_2_N thin films, three major diffraction peaks are noticed. Around 37.1°, a strong diffraction peak appeared and two low intensity peaks are observed around 43.1° and 79.2°, indexed to (111), (200) and (222) crystallographic orientations of Mo_2_N. All diffraction peaks were found to be well matched with γ-Mo_2_N (JCPDS file: PDF # 25-1366) having a cubic crystal system. The intensity of the diffraction peak 37.1° shifted slightly with respect to the higher amount of Cr doping concentration in the Mo_2_N film (Fig. 2b), confirming Cr ions in the Mo_2_N lattice and a sharp intensity peak at 37.1°, indicating high crystallinity of the prepared thin film samples. In Fig. 2b, the diffraction pattern of Cr-doped Mo_2_N, at 2*θ* around 37.1°, shifted to the right  compared to the pristine Mo_2_N. Such a shift in the peak confirms the substitution of Cr in the Mo site with the presence of tensile stress in the as-deposited films [37, 38]. The expanding grain size caused tensile stress in the as-developed films, demonstrating a decreased lattice parameter (4.13 Å) relative to bulk Mo_2_N. In the as-grown films, the XRD pattern exhibited no peaks corresponding to the metallic Mo or Cr, or any other types of Mo/Cr nitrides. By substituting some of the Mo atoms with Cr, it is noted that Cr is completely in a solid solution with Mo_2_N and remains as a cubic structure for the entire Cr dopant concentration in the current investigation. Furthermore, the as-deposited thin films are a pure form of Mo_2_N, whereas the Cr-doped Mo_2_N films are highly crystalline in nature. No secondary phases were detected in the XRD analyses.

### Surface Chemistry Studies

**Fig. 3:**
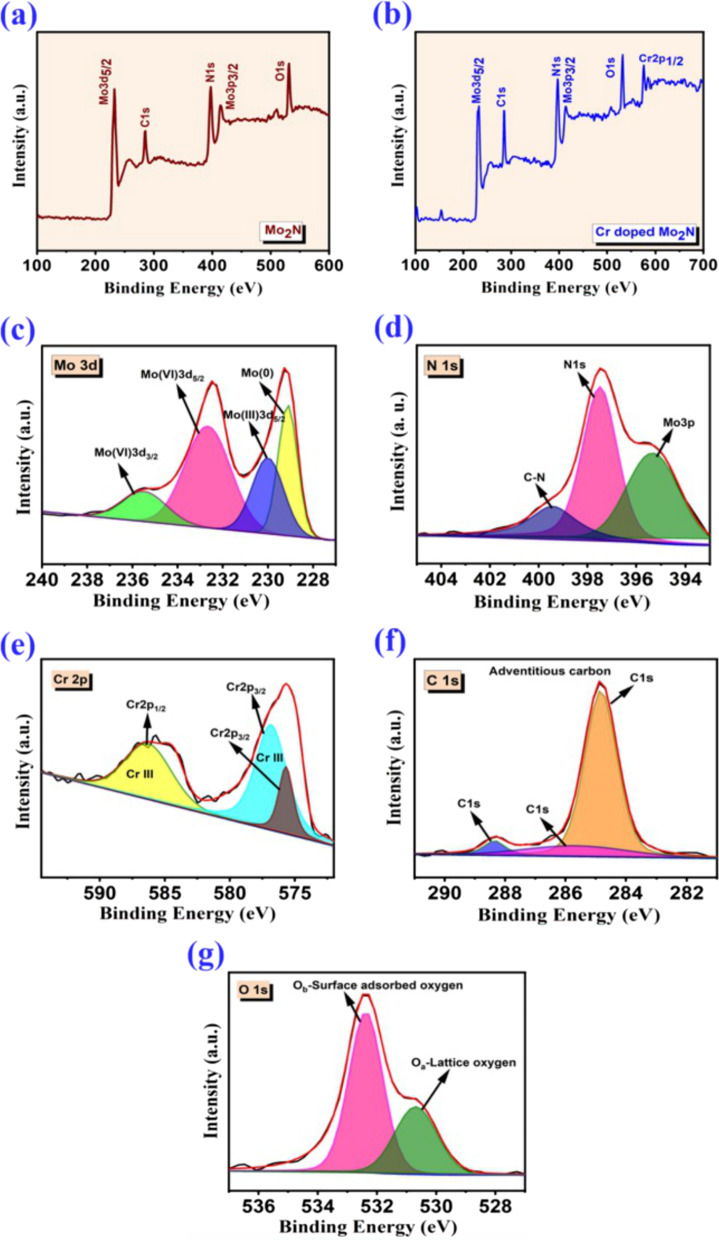
Schema of XPS spectra: **a** un-doped Mo_2_N, **b** Cr-doped Mo_2_N thin films, **c** Mo 3*d*, **d** N 1*s*, **e** Cr 2*p*, **f** C 1*s* and **g** O 1*s*

XPS was applied to the pristine Mo_2_N and Cr/Mo_2_N-4 film samples. The energy-dispersive X-ray spectroscopy technique was used to analyze the bulk composition of coatings. Moreover, the XPS technique can analyze surface chemical states at a depth as far as ~ 5 nm. Figure 3a, b shows the full scan survey spectra of the un-doped Mo_2_N and Cr/Mo_2_N-4 thin films. In Fig. 3c–g, the high-resolution spectra of all the elements present in the thin film samples are displayed. To avoid denitrification of Mo_2_N films due to Ar^+^ bombardment, no Ar^+^ ion etching was done before collecting the X-ray generated electrons. As illustrated in Fig. 3c, the Mo 3d core-level spectrum can be deconvoluted into four peaks, corresponding to Mo^0^ (~ 229 eV), Mo^3+^ (~ 230.1 eV) and Mo^6+^ (232.6 and 235.4 eV) species for the Cr–Mo–N film sample. As shown in Fig. 3f, the impure surface of the adventitious carbon (C 1*s*) at binding energy (BE) of 284.8 eV is denoted [39]. In Fig. 3g, the presence of oxide shows that the surface of the Mo species has been oxidized, indicating both lattice oxygen (O_a_-530.5 eV) and surface observed oxygen (O_b_-532.4 eV), which is mainly due to the contamination by surface oxygen upon exposure to air [40]. Further, two peaks at around 575.7 eV (Cr 2*p*_3/2_) and 586.4 eV (Cr 2*p*_1/2_) are revealed in the Cr-doped Mo_2_N thin film samples [41] and ascribed to Mo–Cr, demonstrating that Cr has been incorporated into the Mo_2_N host lattice.

In the XPS spectra, tiny peaks at 415 eV (Mo_2_N sample, Fig. 3a) and 413.7 eV (Cr/Mo_2_N-4 sample, Fig. 3b) are found. Such peaks are associated with the Mo 3p_3/2_ region. As shown in Fig. 3a, b, the positions of Mo 3d peaks for Mo–N and Mo–O bonds have shifted somewhat toward the low binding energy area. This behavior can be due to Cr doping, which can lower their bonding energy by sharing the Mo binding connection. In addition, one more primary peak can be detected for both Mo–N and Cr–Mo–N in the N 1*s* XPS spectra; the Mo–N bond is responsible for the peak at 397.4 eV (Fig. 3d). The N 1*s* peak for Cr–Mo–N has shifted to 396.8 eV (397.4 eV for the bare Mo–N), implying that the Cr element has been doped into the Mo_2_N lattice by replacing one of the Mo/N elements. In Fig. 3e, the Cr 2*p* XPS spectrum of Cr–Mo–N is seen to have a major peak at 575.7 eV. This peak differs significantly from the 574.2 eV of the Cr metal and the 576.0 eV of Cr, suggesting that the Cr dopants in the Mo–N lattice are Cr ions. In addition, the atomic percentages of the elements present in the thin film samples were roughly calculated from the XPS spectra as follows: Mo-33.18%, N-27.36%, C-15.72%, Cr-9.48% and O-14.26% for the Cr-doped Mo_2_N sample and Mo-39.96%, N-33.07%, O-12.39% and C-14.58% for Mo_2_N, respectively.

### Morphological Studies

**Fig. 4:**
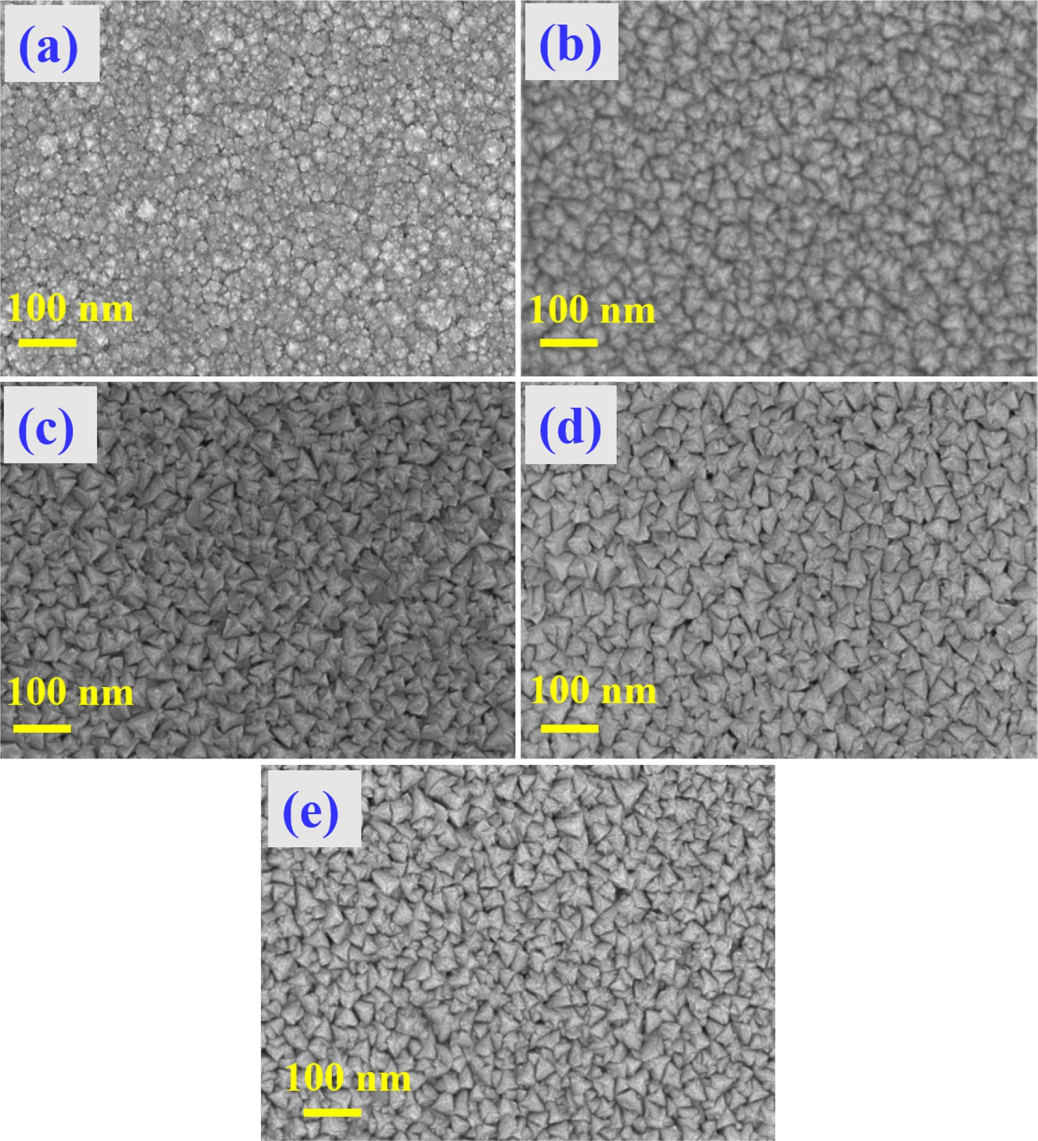
FE-SEM images: **a** Mo_2_N, **b** Cr/Mo_2_N-1, **c** Cr/Mo_2_N-2, **d** Cr/Mo_2_N-3 and **e** Cr/Mo_2_N-4 thin films

Morphological transformation of the pristine Mo_2_N and Cr-doped Mo_2_N thin films produced by the reactive co-sputtering process was evaluated using FE-SEM analysis. In Fig. 4a–e, FE-SEM images of the un-doped Mo_2_N and Cr-doped Mo_2_N thin films samples prepared at different concentrations of Cr doping are displayed, respectively. In Fig. 4a, the pure Mo_2_N thin film sample exhibits an agglomerated granular microstructure having a smooth surface; average particles are 20–30 nm in size. When doping concentration increases, all Cr/Mo_2_N thin film samples signify that the particles are densely packed together and are spread uniformly over the surface (Fig. 4b–e). The FE-SEM images of the Cr-doped Mo_2_N samples reveal a triangular pyramidal like surface morphology [42, 43].

### Elemental Composition Studies

**Fig. 5:**
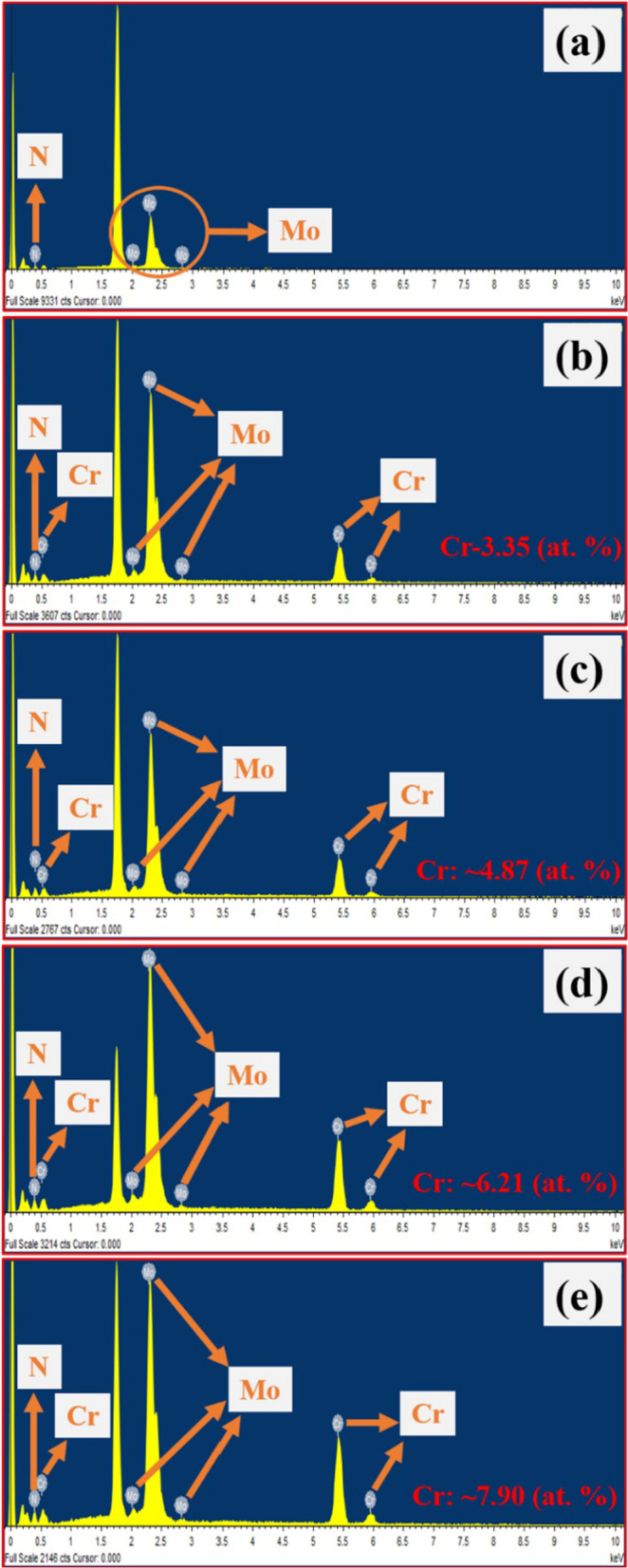
EDS elemental analysis: **a** Mo_2_N, **b** Cr/Mo_2_N-1, **c** Cr/Mo_2_N-2, **d** Cr/Mo_2_N-3 and **e** Cr/Mo_2_N-4 thin films

To examine both the elemental distribution and formation of Mo_2_N and Cr-doped Mo_2_N thin films, FE-SEM and EDS analyses were carried out. In Fig. 5, EDS reports of the as-prepared thin film samples are depicted. In Fig. 5a, the EDS spectra, viz. the Mo and N signals, can be seen for the un-doped Mo_2_N. In Fig. 5b–e, Cr, Mo and N signals are found in the Cr-doped Mo_2_N thin films. As Cr doping concentration increased, the intensity of the Cr peak increased**,** confirming the successful formation of the Cr-doped Mo_2_N thin films. In Fig. 5, the atomic percentiles for the Cr-doped Mo_2_N samples are shown. Besides, the EDS elemental color mapping images of Mo_2_N and Cr-doped Mo_2_N TFEs are highlighted, which verify the uniform deposition and distribution of Mo and N elements in the Mo_2_N thin film sample. Mo, Cr and N elements are all present in the Cr-doped Mo_2_N thin film samples (Additional file 1: Fig. S3).

### Electrochemical Supercapacitor Performances

Electrochemical performance of the pristine Mo_2_N and Cr-doped Mo_2_N binder-free TFEs was carried out in 1 M KOH aqueous electrolyte under room temperature in a three-electrode cell setup. The applied voltage window of − 1.2 to − 0.2 V was fixed for both CV and GCD analysis.

#### Cyclic Voltammetry Analysis

**Fig. 6:**
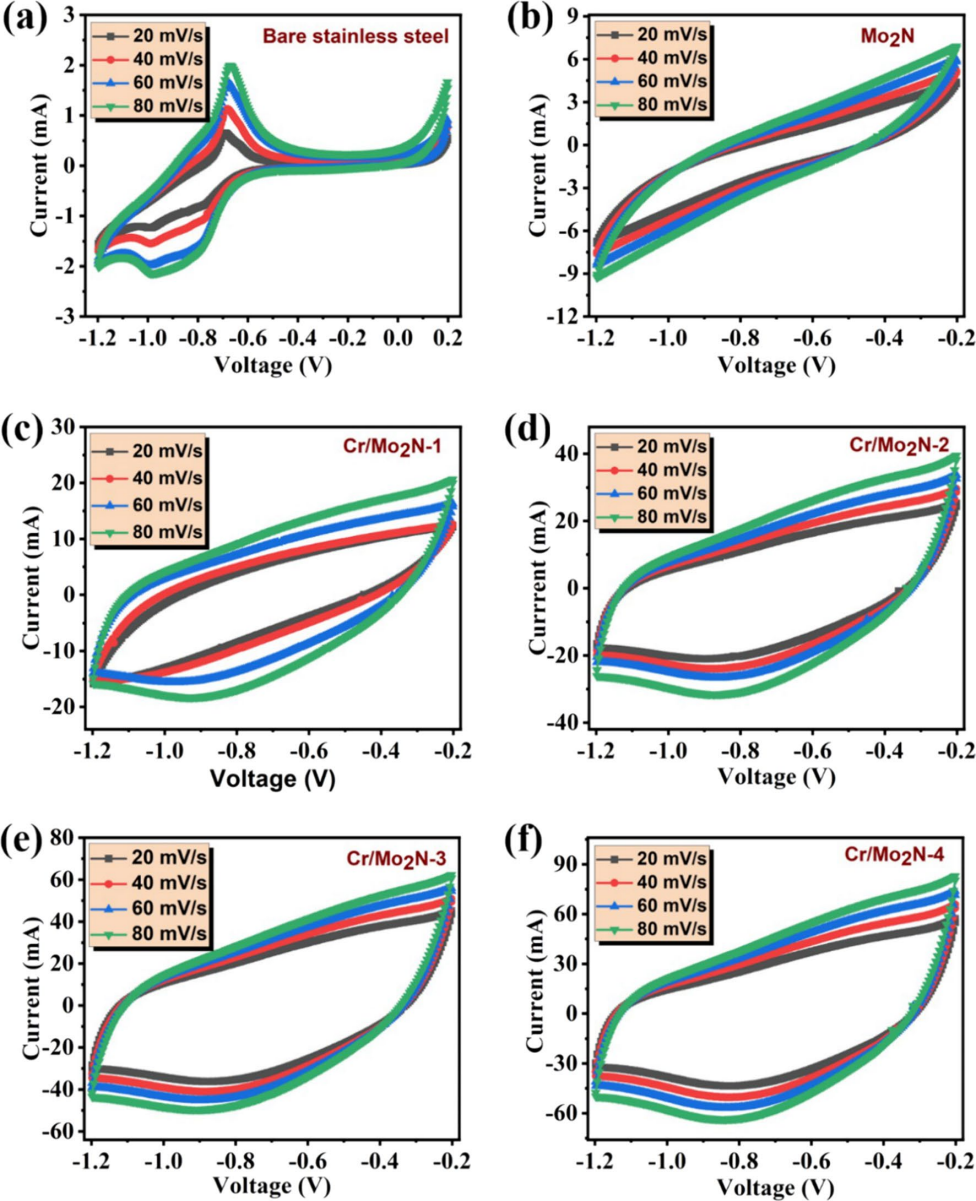
Cyclic voltammograms: **a** bare stainless steel substrate, **b** as-deposited Mo_2_N and **c**–**f** Cr-doped Mo_2_N TFEs measured at different scan rates (10–100 mV/s)

**Fig. 7:**
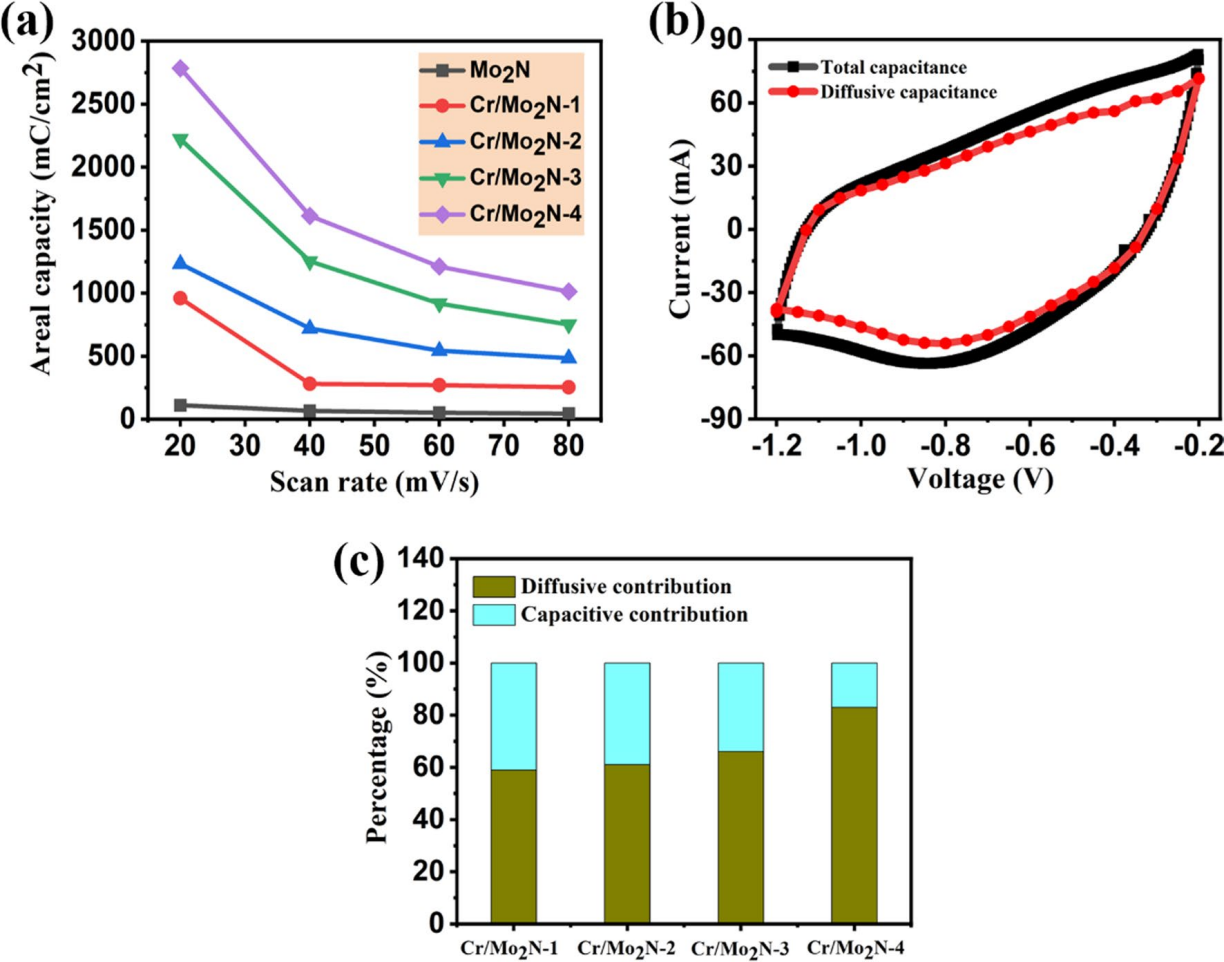
**a** Areal capacities for the Cr-doped Mo_2_N tested under various scan rates, **b** plot of diffusion and capacitive contribution of Cr/Mo_2_N-4 electrode evaluated, at a scan rate of 80 mV/s, and **c** illustrating the diffusive and capacitive contributions of different doping concentration of Cr-doped Mo_2_N electrodes, at a fixed scan rate of 80 mV/s

The as-deposited Mo_2_N and Cr-doped Mo_2_N TFEs were further characterized via CV analysis under different scan rates (20–80 mV/s). Initially, both the stability and reversibility of the electrodes were examined, applying 10 CV cycles at a fixed scan rate of 50 mV/s. In Fig. 6, the CV curves of the bare stainless steel substrate, as-deposited pristine Mo_2_N and Cr-doped Mo_2_N TFEs are displayed. The shape of the CV curves for all the Cr-doped Mo_2_N TFEs is almost similar, and the higher doping concentration of Cr/Mo_2_N-4 electrode exhibits the bigger CV area compared to pristine Mo_2_N indicating the excellent capacity behavior, outstanding reversibility and rate capability [34]. It is also noted that the CV curves of the metal nitride-based electrodes revealed a quasi-rectangular shape having a superior active surface area even at lower scan rates, suggesting excellent charge storage behavior and high-rate capability [44–47]. The areal capacity of the Mo_2_N and Cr-doped Mo_2_N-based TFEs can be calculated from the CV curves:

1 $${\mathrm{Areal}}\, {\mathrm{capacity}}\, ({Q}_{\mathrm{a}})=\frac{I}{A\times \nu }$$

where *Q*_a_ is the areal capacity (mC/cm^2^), *I* is the current (A), *A* is the exposed active area of the electrode (cm^2^), and *ν* is the scan rate (mV s^−1^). Thus, via CV analysis, the maximum areal capacities of Cr/Mo_2_N-4 are found to be 2780 mC/cm^2^, Cr-doped Mo_2_N-3: 2220 mC/cm^2^, Cr-doped Mo_2_N-2: 1233 mC/cm^2^, Cr-doped Mo_2_N-1:960 mC/cm^2^ at the scan rate of 20 mV/s. The measured areal capacities of the battery-type Cr-doped Mo_2_N electrodes are remarkably greater than those of the un-doped Mo_2_N thin film electrode (110 mC/cm^2^), demonstrating the superior charge storage performance of the binder-free electrodes made of other metal nitrides [48, 49]. The increased areal capacity of the grown Cr-doped Mo_2_N TFEs may well be caused by the electrolyte ions' increased mobility at the interface between the aqueous electrolyte and active electrode, as well as the synergetic contribution of both Cr and Mo_2_N. In Fig. 7a, it is noted that the areal capacity values were found to be substantially greater than those of other metal nitrides-based electrodes (VN, CrN, TiN, etc.) that had previously been published. In Table 2, the performance of the electrodes are summarized.

**Table 2:** Comparison of the electrochemical performance of Cr-doped Mo_2_N film electrodes with other reported metal nitride-based electrode materials

No.	Materials	Synthesis method	Electrolyte	Areal or specific capacitance/capacity	Capacitance/capacity retention with number of cycles	References
1	TiN	Direct current magnetron sputtering	0.5 M K_2_SO_4_	146.4 F cm^−3^	–	[15]
2	RuN	Reactive sputtering	1 M LiPF_6_	37 F/g	–	[20]
3	Ni_2_Mo_3_N	Ammonolysis synthesis	6 M KOH	264 C/g	81.4%@1000 cycles	[24]
4	Ni_3_N	Radio-frequency magnetron sputtering	3 M KOH	319 mF cm^−2^	93.7%@2000 cycles	[25]
5	γ-Mo_2_N	DC sputtering	0.5 M Li_2_SO_4_	722 F/cm^3^	100%@2000 cycles	[33]
6	MoN_*x*_@NF	Atomic layer deposition (ALD)	1 M KOH	130 mC/cm^2^	100%@8000	[48]
7	Ni-Co_4_N@NC	In situ nitridation process	1 M KOH	397.5 mAh/g	72.4%@10,000 cycles	[50]
8	Mo_3_N_2_	Reactive magnetron co-sputtering	1 M KOH	173.4 mF cm^2^	–	[51]
9	Cu/Mo_3_N_2_	Reactive magnetron co-sputtering	1 M KOH	619.5 mF cm^2^	80%@2000 cycles	[51]
10	Ni-doped Co–Co_2_N	Chemical synthesis	1 M KOH	361.93 C/g	82.4%@5000 cycles	[52]
11	W_2_N (thick coating)	Sputtering technique	1 M KOH	0.55 F cm^−2^ 700 F cm^−3^	10,000 cycles	[53]
12	MoN/TiN	Electrodeposition/nitridation treatment in NH_3_	1 M LiOH	121.50 mF cm^−2^	93.8%@1000 cycles	[54]
13	VN	DC plasma reactive sputtering	1 M KOH	238.2 mF cm^−2^	77.5%@2000 cycles	[55]
14	Ni_0.2_Mo_0.8_N	Facile and simple nitridation process	6 M KOH	2446 mC/cm^2^	80.1%@6000 cycles	[56]
15	Mn_3_N_2_	Direct current magnetron sputtering	Different electrolytes	118, 68 & 27 mF cm^−2^	98.5% (KOH), 89% (KCl) and 83% (Na_2_SO_4_) @4000 cycles	[57]
16	CrN	Direct current magnetron sputtering	0.5 M H_2_SO_4_	40.53 mF cm^−2^	95.3%@2000 cycles	[58]
17	CrN	Direct current magnetron sputtering	0.5 M Na_2_SO_4_	32.69 mF cm^−2^	93.8%@2000 cycles	[58]
18	CrN	Direct current magnetron sputtering	0.5 M NaCl	9.17 mF cm^−2^	89.9% 93.8%@2000 cycles	[58]
19	TiN/C	Reactive sputtering	0.5 M H_2_SO_4_	45.81 mF cm^−2^	85%@5000 cycles	[59]

Moreover, to better understand the charge storage behavior of Cr-doped Mo_2_N TFEs via CV analysis, Dunn’s approach is adopted to recognize the two different charge storage contributions: (i) surface-controlled and (ii) diffusion-controlled, which states that the total current at a given potential is the sum of the diffusive and capacitive currents investigated [60]:

2 $$i(V) = k_{1} \nu + k_{2} \nu^{0.5}$$

3 $$i(V)/\nu^{0.5} = k_{1} \nu^{0.5} + k_{2}$$

where *k*_1_ and *k*_2_ are differentiated as surface- and diffusion-controlled contributions of the developed TFEs, respectively, *i*(*V*) is the current of the given potential, ν is the applied scan rate, and *k*_1_ and *k*_2_ values are calculated by plotting the graph between and *i*(*V*)/*ν*^0.5^ and (*ν*)^0.5^. In Fig. 7b, the CV curves of the diffusive and capacitive contribution of the Cr-doped Mo_2_N-4 electrode evaluated at a scan rate of 80 mV/s are shown, revealing ~ 83% diffusive contribution to accumulate the charge of the electrode. Besides, the diffusive contribution of Cr/Mo_2_N-1, Cr/Mo_2_N-2, Cr/Mo_2_N-3 and Cr/Mo_2_N-4 TFEs is shown to be ~ 83%, ~ 66%, ~ 61% and ~ 59%, respectively (Fig. 7c). These findings suggest that the as-developed nitride-based electrodes have excellent charge storage behavior, with almost mixed contributions (capacitive and diffusive) to total charge storage for lower doping concentrations of Cr in Mo_2_N electrodes and the maximum diffusive contribution behavior observed for the higher doping percent of Cr in Mo_2_N. As a result, the Cr-doped Mo_2_N electrodes are considered to be potential candidates for high-performance energy storage device applications due to the synergetic contribution between Cr dopant and Mo_2_N. Additionally, the mechanism of the electrochemical reaction between the active electrode material and the electrolyte can be described as follows:

4 $${\text{Mo}}_{x} {\text{N}} + {\text{OH}}^{ - } \leftrightarrow {\text{Mo}}_{x} {\text{NOH}} + {\text{e}}^{ - }$$

#### Galvanostatic Charge–Discharge Analysis

**Fig. 8:**
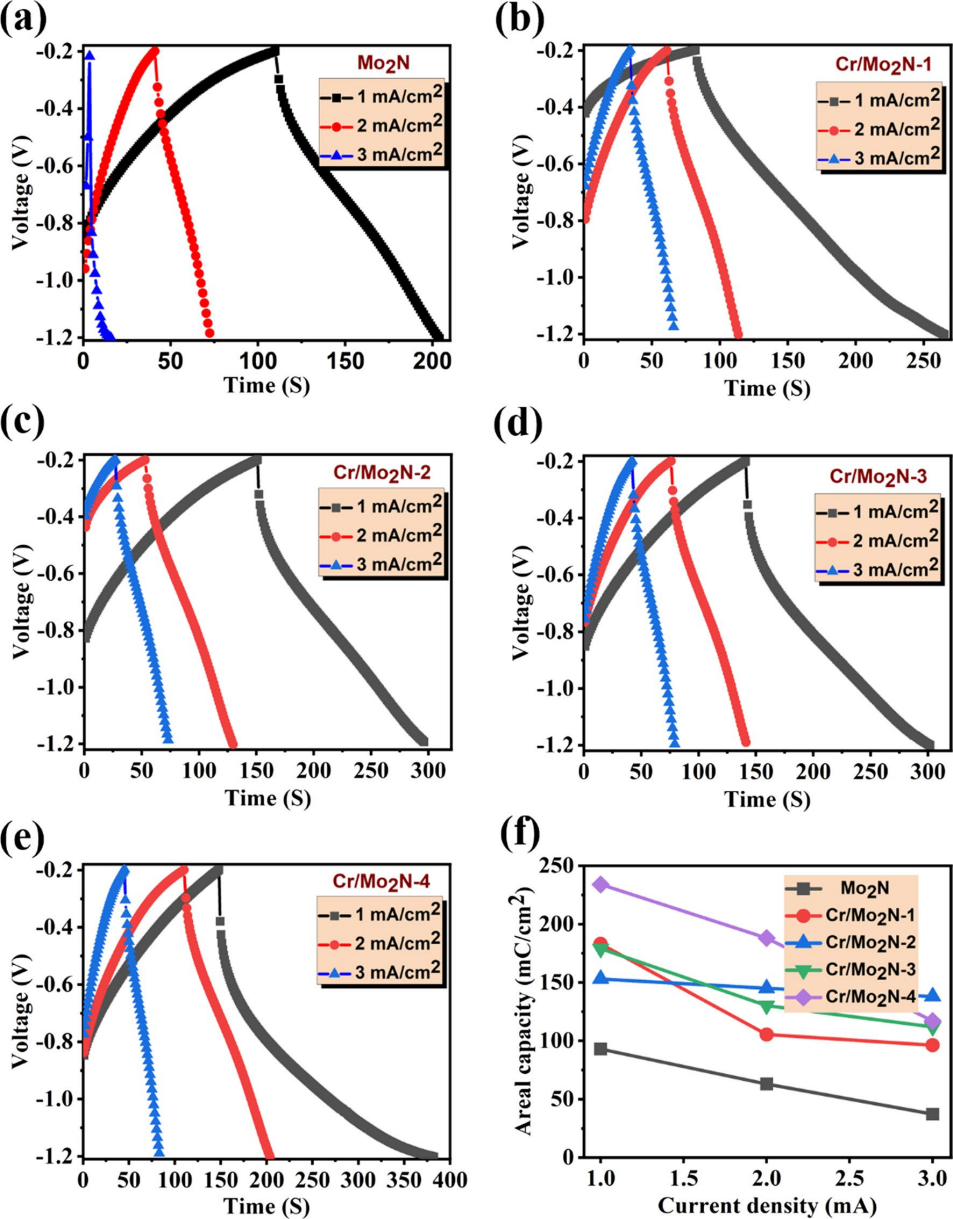
Charge–discharge profiles: **a** pristine Mo_2_N, **b** Cr/Mo_2_N-1 TFE, **c** Cr/Mo_2_N-2 TFE, **d** Cr/Mo_2_N-3 TFE, **e** Cr/Mo_2_N-4 TFE and **f** areal capacity with respect to different current densities (1–3 mA/cm^2^)

The charge–discharge (CD) performance of the as-prepared electrodes was further examined via GCD studies in 1 M KOH aqueous electrolyte. In Fig. 8a–e, the GCD profiles of Mo_2_N and Cr-doped Mo_2_N TFEs conducted at different current densities ranging from 1 to 3 mA/cm^2^ in a fixed voltage window of − 1.2 to − 0.2 V are presented. In the GCD analysis, the nonlinear shape of the charge–discharge profiles is clearly visible, indicating an ideal capacitive nature; a similar trend of charge–discharge patterns has been seen in previous studies [45, 61]. Hence, the energy storage in the Cr-doped Mo_2_N TFEs is attributed to both physisorption of the electric double layer (EDL) and faradaic reaction. The Cr-doped Mo_2_N electrode shows a dramatically longer discharging time compared to the pristine Mo_2_N electrode, which may account for the substitution of Cr in the Mo_2_N lattice. The areal capacity of the TFEs, according to the GCD analysis, is obtained for various current densities and can be expressed as:

5 $${\mathrm{Areal}}\, {\mathrm{capacity}}\, ({Q}_{\mathrm{a}})=\frac{I\times \Delta t}{A}$$

where *Q*_a_ is the areal capacity (mC/cm^2^), *I* is the current (A), Δ*t* is the time difference between the charge/discharge profile (s), and *A* is the exposure active area of the electrode (cm^2^).

**Fig. 9:**
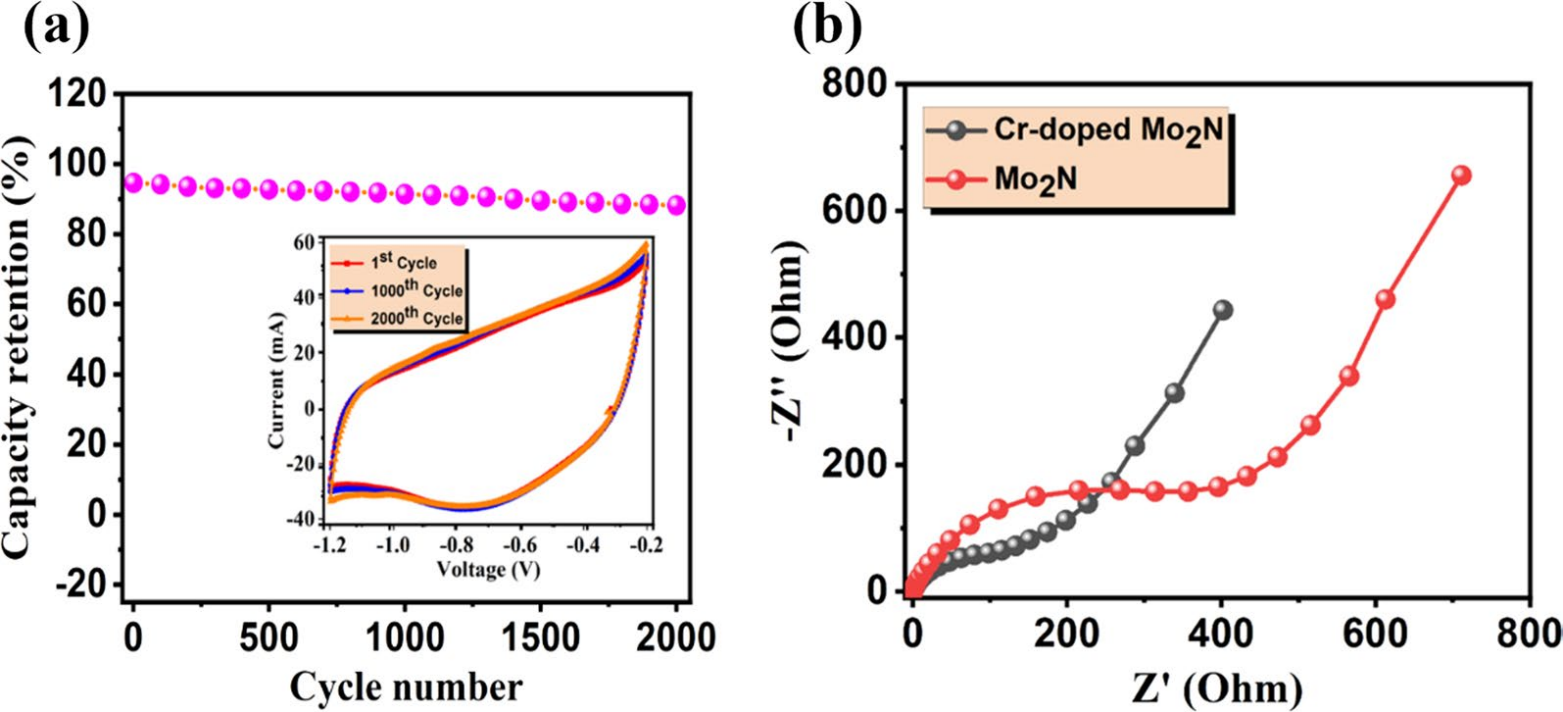
**a** Cycling performance of Cr-doped Mo_2_N-4 thin film electrode performed at 60 mV/s for 2000 cycles (inset: depicting the CV curves of 1st, 1000th and 2000th cycles) and **b** Nyquist plot of un-doped and Cr-doped Mo_2_N TFEs

Based on the GCD results, the maximum areal capacity of 243 mC/cm^2^ for the Cr-doped Mo_2_N and 93 mC/cm^2^ for the un-doped Mo_2_N was attained in 1 mA/cm^2^ current density. All the Cr-doped Mo_2_N TFEs demonstrated high areal capacities more than the un-doped Mo_2_N TFEs (Fig. 8f). These results proved to be much higher than previously reported nitride-based electrodes: CrN [36], Mn_3_N_2_ [57], Co_3_N [62], Nb_4_N_5_ [63], TiN [64], HfN [65], GaN [66], VN [67] and W_2_N [68]. As doping concentration of Cr increased, areal capacity values increased, with respect to various current densities. In Fig. 9f, it is clear that when current density increased, areal capacities decreased, due to insufficient time for complete ion exchange in the electrolyte/electrode interface at higher current densities [35]. CD profiles show a curvy and symmetrical linear shape and indicate the superior charge storage behavior of the electrodes having low ohmic potential loss (IR drop), reflecting the great capacity and reversibility of the electrodes [55, 69]. Such positive CD characteristics arose owing to the synergistic effect of doping Cr with the Mo_2_N electrode. The high-efficiency behavior of the as-prepared Cr-doped Mo_2_N thin film-based electrodes verifies their potential for use as high-performance ECs.

#### Cycling Stability and Impedance Analysis

Cycling behavior and rate performance are essential parameters for the practical application of energy storage devices. In Fig. 9a, the Cr/Mo_2_-4 electrode measured up to 2000 CV cycles, demonstrating maximum capacity retention of ~ 94.6%. As the number of cycles increased (up to 2000), the rate capability of the electrodes is seen to decrease slowly, which indicates that the prepared nitride-based electrodes are long-lasting even at higher cycles: significantly higher than previously studied metal nitrides such as Nb_4_N_5_ [46], VN [70] and TiN [71]. It is acknowledged that the improved electrochemical performance of the Cr-doped Mo_2_N electrodes can be attributed to the synergistic effect between the dopant and host materials.

Finally, to understand electrochemical kinetics such as the charge transfer process between as-prepared nitride-based electrodes and electrolyte interface, EIS studies were carried out. In Fig. 9b, it is found that the Nyquist plot of Mo_2_N and Cr-doped Mo_2_N TFEs performed at a frequency range of 1 Hz–100 MHz with an amplitude of 10 mV. According to the electrochemical Nyquist plot, the curve can be separated into three components: electrolyte resistance, a vertical line in the low-frequency zone and a semicircle in the high-frequency zone. The electrolyte resistance, namely the ionic resistance of the electrolyte, intrinsic resistance of the electrode and interface resistance, is represented by the intersection with the x-coordinate in the high-frequency region [72]. The vertical/inclined line in the low-frequency zone of as-prepared nitride-based electrodes is due to the diffusion of ions at the electrode–electrolyte interface, indicating the remarkable conductive behavior of the electrode [34]. The semicircle in the high-frequency zone represents charge transfer resistance (*R*_ct_) in the electrode and electrolyte interface along with the *R*_ct_ of 521 Ω for the un-doped Mo_2_N sample and 130 Ω for the Cr-doped Mo_2_N thin film sample. During the electrochemical process, the decrease in charge transfer resistance after doping Mo_2_N with Cr may be due to rapid electron and ion transfer and excellent electrolyte accessibility. Such an outcome demonstrates that the Cr-doped Mo_2_N TFEs can increase the electrode reaction kinetics of binder-free ECs.

## Conclusions

In summary, the nanopyramidal-shaped Cr-doped Mo_2_N binder-free TFEs were prepared via a reactive co-sputtering technique; their microstructural and electrochemical energy storage properties were systematically elucidated. It is significant that the doping effect of the Cr transition metal played an important role in enhancing the electrochemical energy storage performance of the Mo_2_N TFEs. The obtained un-doped and Cr-doped Mo_2_N thin films were investigated in detail via XRD, XPS, FE-SEM with EDS analyses. CV studies demonstrated that the ~ 7.9 at.% Cr-doped Mo_2_N TFE exhibited a maximum areal capacity of 2780 mC/cm^2^, which proved to be much greater than the pristine Mo_2_N electrode and other nitride-based TFEs shown in prior investigations. Furthermore, the GCD study demonstrated that the charge–discharge profiles have a symmetrical linear shape, exhibiting outstanding discharge behavior, with a areal capacity of 243 mC/cm^2^. The higher doping concentration of Cr-doped Mo_2_N electrode displayed outstanding cycling stability, with a capacity loss of only 5.4% after 2000 CV cycles. The nitride-based TFEs prepared by the reactive co-sputtering technique were found to be a simple technique for developing high-performance binder-free electrodes that proved to have excellent cycling stability, displaying superior electrochemical characteristics for highly valued future energy storage devices.

## Supplementary Information

**Additional file 1.** Growth mechanism of Cr doped Mo_2_N TFEs through sputtering technique; FESEM and EDS mapping of TFEs.

## Data Availability

The datasets used and/or analyzed during the current study are available from the corresponding author on reasonable request.
