# Supplementary material for: Sputter-Deposited Binder-Free Nanopyramidal Cr/γ-Mo2N TFEs for High-Performance Supercapacitors
Source: Nanoscale Res Lett. 2022 Jul 19;17:65. doi: 10.1186/s11671-022-03704-5 (PMC9296755; doi:10.1186/s11671-022-03704-5)
Supplement: Supplementary file 1 — Additional file 1. Growth mechanism of Cr doped Mo2N TFEs through sputtering technique; FESEM and EDS mapping of TFEs. [file 11671_2022_3704_MOESM1_ESM.docx]

**Supporting information:**

**Sputter Deposited Binder-Free** **Nanopyramidal Cr/****γ-Mo_2_N Thin Film Electrodes for High Performance Supercapacitors**

Durai Govindarajan^a^, Nithyadharseni Palaniyandy^b^, Karthik kumar Chinnakutti^c^, Mai Thanh Nguyen^d^, Tetsu Yonezawa^d^, Jiaqian Qin^e^, Soorathep Kheawhom^a, f, g,^*

***^a^****Department of Chemical Engineering, Faculty of Engineering, Chulalongkorn University, Bangkok 10330, Thailand.*

***^b^****Institute for the Development of Energy for African Sustainability, College of Engineering, Science and Technology, University of South Africa, Florida Science Campus, Roodepoort 1709, South Africa.*

***^c^****Department of Chemistry, Vinayaka Mission’s Kirupananda Variyar Arts and Science College, Vinayaka Mission’s Research Foundation (Deemed to be University), Salem-636308, India.*

***^d^****Division of Materials Science and Engineering, Faculty of Engineering, Hokkaido University, Hokkaido 060-8628, Japan.*

*^e^Metallurgy and Materials Science Research Institute, Chulalongkorn University, Bangkok 10330, Thailand.*

***^f^****Center of Excellence on Advanced Materials for Energy Storage, Chulalongkorn University, Bangkok 10330, Thailand.*

***^g^****Bio-Circular-Green-economy Technology & Engineering Center (BCGeTEC), Faculty of Engineering, Chulalongkorn University, Bangkok 10330, Thailand.*

^*^Corresponding author Email: [soorathep.k@chula.ac.th](mailto:soorathep.k@chula.ac.th)


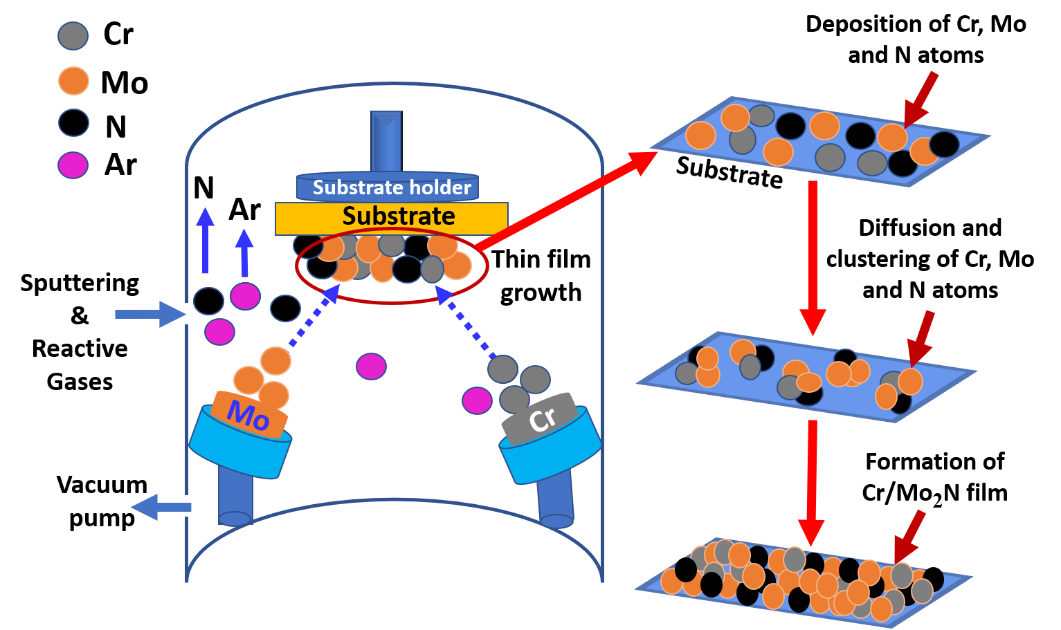


**Figure S1.** Growth mechanism of Cr doped Mo_2_N thin films through sputtering techniqe.


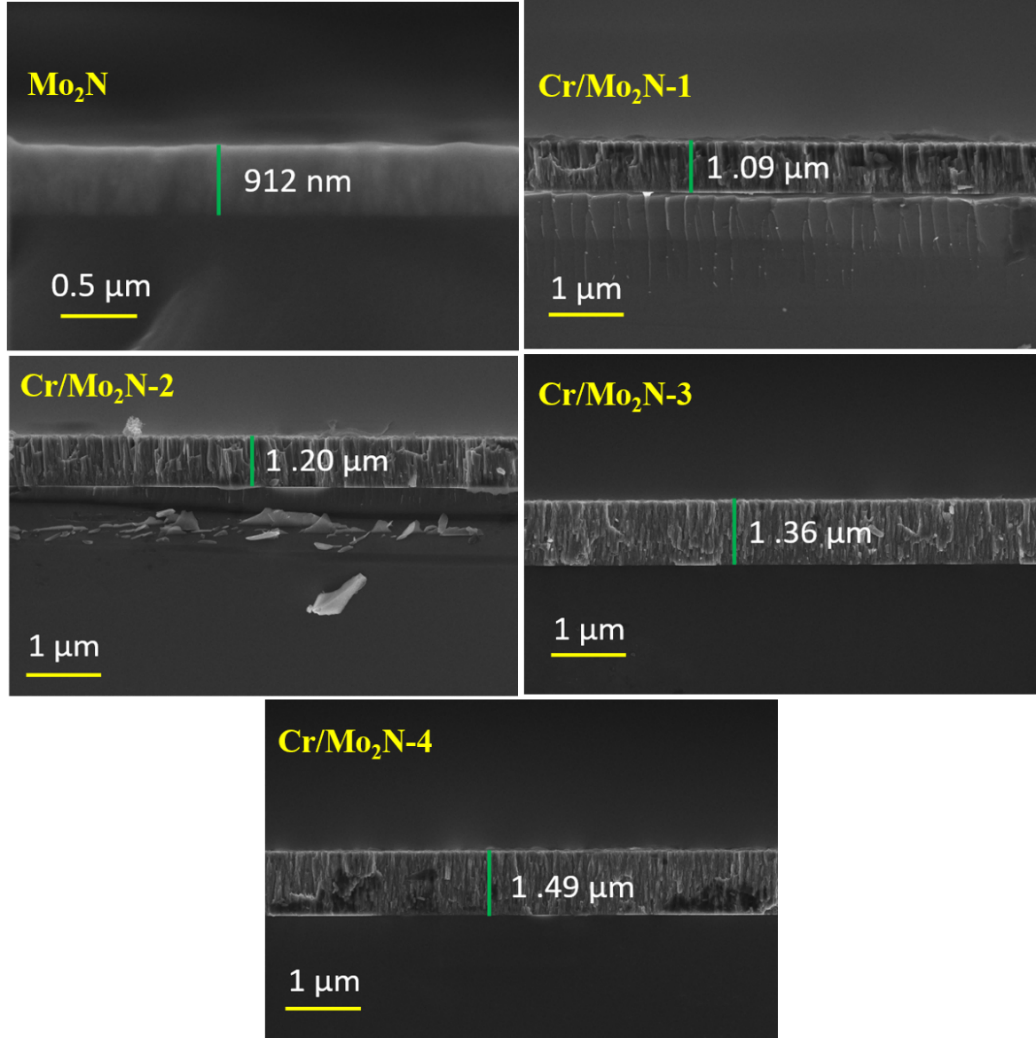


**Figure S2.** FE-SEM cross-sectional images of the as-deposited films.


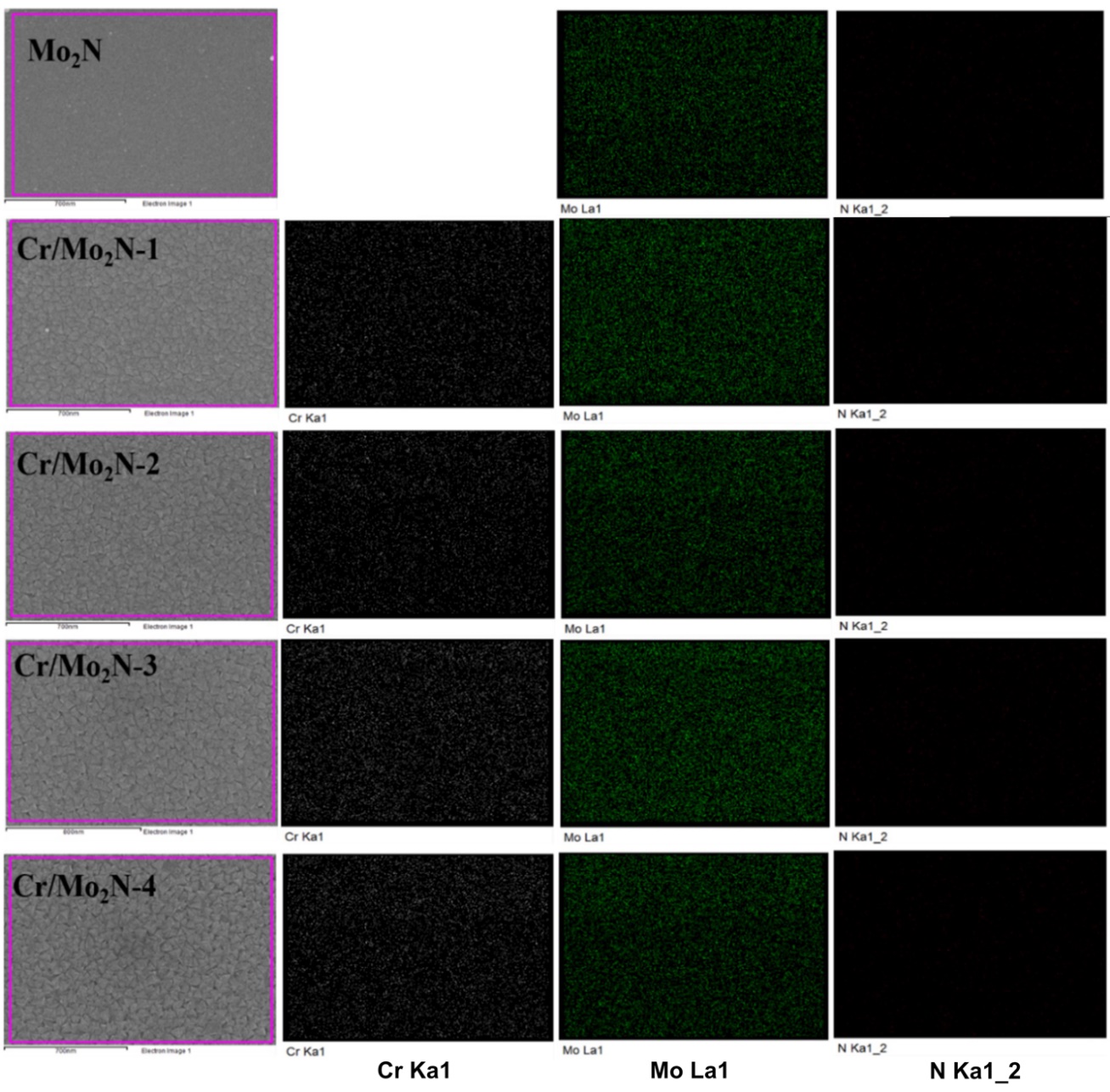


**Figure S3.** FE-SEM and their corresponding EDS mapping elemental images of as-deposited Mo_2_N and Cr doped Mo_2_N thin films.
